# The Versatile Manipulations of Self-Assembled Proteins in Vaccine Design

**DOI:** 10.3390/ijms22041934

**Published:** 2021-02-16

**Authors:** Que Dan Nguyen, Kosuke Kikuchi, Basudev Maity, Takafumi Ueno

**Affiliations:** Department of Life Science and Technology, Tokyo Institute of Technology, Nagatsuta-cho, Midori-ku, Yokohama 226-8501, Japan; nguyen.d.ag@m.titech.ac.jp (Q.D.N.); kikuchi.k.aq@m.titech.ac.jp (K.K.); basudev@bio.titech.ac.jp (B.M.)

**Keywords:** protein assemblies, virus-like particles, non-viral proteins, vaccine applications, subunit vaccines

## Abstract

Protein assemblies provide unique structural features which make them useful as carrier molecules in biomedical and chemical science. Protein assemblies can accommodate a variety of organic, inorganic and biological molecules such as small proteins and peptides and have been used in development of subunit vaccines via display parts of viral pathogens or antigens. Such subunit vaccines are much safer than traditional vaccines based on inactivated pathogens which are more likely to produce side-effects. Therefore, to tackle a pandemic and rapidly produce safer and more effective subunit vaccines based on protein assemblies, it is necessary to understand the basic structural features which drive protein self-assembly and functionalization of portions of pathogens. This review highlights recent developments and future perspectives in production of non-viral protein assemblies with essential structural features of subunit vaccines.

## 1. Introduction

Self-assembled proteins with variable nanoscale structures such as cages, tubes, wires, and rings have been used as platforms for applications in a wide range of biomolecular engineering efforts and biomedical applications [1,2,3]. Due to their unique structural features, symmetry and relative ease of modification, such proteins are versatile carriers for delivery of molecules such as drugs and imaging agents and can be easily functionalized with proteins, peptides, and synthetic molecules [1,3,4]. Development of vaccine candidates based on protein assemblies is a powerful strategy because protein assemblies can be easily fused with portions of inactivated pathogens or antigens to generate a safe molecular entity which can be effectively delivered into cells to induce immune responses.

**Figure 1:**
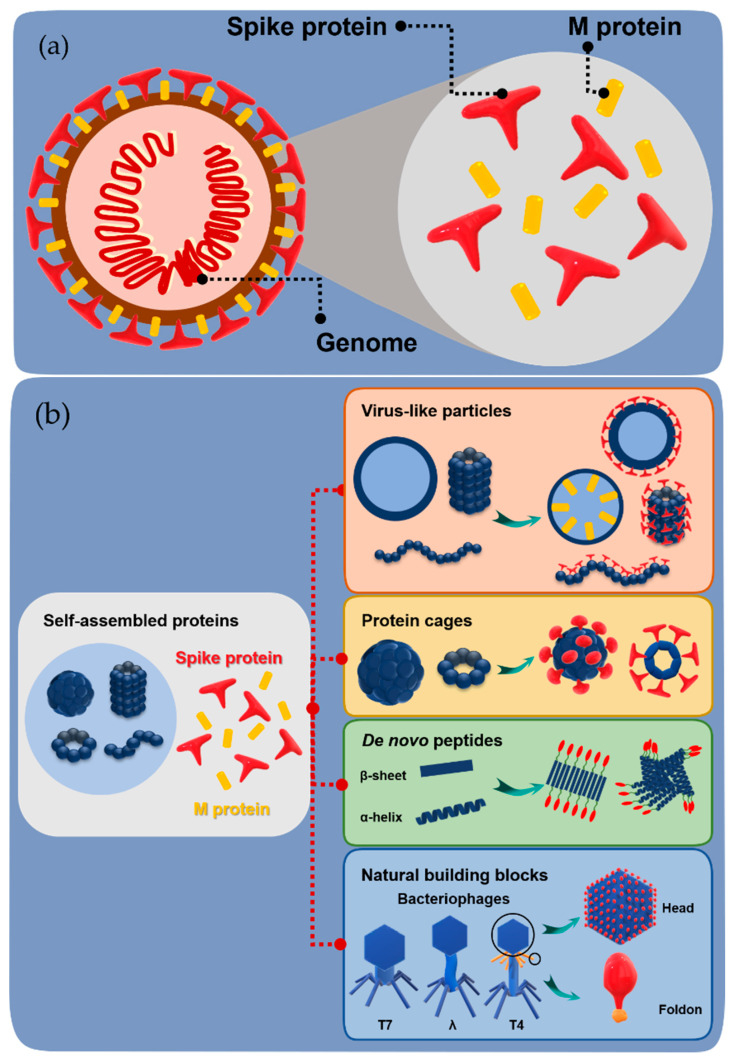
General structure of a pathogen and construction of subunit vaccines. (**a**) Schematic representation of the basic structure of a virus containing spike and membrane protein (M protein) on the surface and the genome inside. (**b**) Schematic representation of the construction of subunit vaccines by fusion of spike/M protein with various self-assembled proteins.

Traditional vaccines are based on live-attenuated or inactivated pathogens. These entities have risk factors such as allergies, off-target responses, side effects, and short-term or insufficient protection across mutating and complex pathogen strains [5,6]. To avoid such issues, subunit vaccines have been developed which contain only parts of pathogens or antigens (Figure 1a). These vaccines are recognized by cells as foreign proteins and immune responses are triggered. Such vaccines are safer, simpler, and can be more efficiently produced than vaccines based on inactivated pathogens despite the fact that they have more advantages than disadvantages. Although subunit vaccines can induce generation of memory helper T cells and B cells, memory killer T cells are not generated because antigen presenting cells (APCs) are not infected [7]. This leads to insufficient long-lasting protection in comparison to traditional vaccines. Thus, to improve vaccine efficiency, one approach, which is particularly useful for small antigens (<10 nm), is to form larger particles or aggregates with adjuvants to promote the immune response to antigens [5,8,9]. Another approach for subunit vaccine development is to modify carrier materials via biomolecular engineering [5,10,11]. In such an approach, the spike or membrane proteins of a pathogen are displayed on the surface of a carrier or encapsulated within a carrier.

In relatively recent developments, a range of protein and peptide biomaterials have been used to increase the protective potency of subunit vaccines for prevention of infectious diseases as well as cancer and autoimmune diseases. The common carrier molecules used in engineered subunit vaccines are either virus-like particles or non-viral proteins such as, de novo peptides or natural building blocks (Figure 1b). Although there are several articles summarizing the development of protein-based vaccines, it is necessary to understand basic criteria for selection of carriers such as shape, surface charge, hydrophobicity, and size, which are very important for effective interaction of the carriers with APCs [12,13,14]. The size of biomaterial carriers and antigens influence the uptake, processing, and transport of antigens, as well as B cell (humoral immunity) and dendritic cell targeting [9,13]. In this context, this review highlights significant recent advances in design of non-viral self-assembled protein carriers for engineered subunit vaccines and perspectives in using such carriers as building blocks for construction of promising vaccine candidates.

## 2. Virus-Like Particles (VLPs)

**Figure 2:**
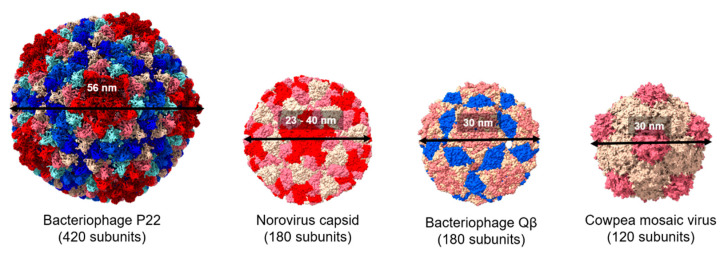
Comparison of the structure of viral protein cages. Space filling models showing the structure of bacteriophage P22, norovirus capsid, cowpea mosaic virus, and bacteriophage Qβ (Protein Data Bank IDs: 2XYZ, 1IHM, 1NY7, and 1QBE, respectively). Subunit chains are distinguished by different colors. All the structures were generated using UCSF ChimeraX.

An interesting and useful aspect of virus-like particles in the context of vaccine development is that they are particulate and have repetitive structures which can provide arrays of the immunogenic antigens required for the induction of a potent antibody response. VLPs are thus one of the most common platforms used in vaccine agents used in prevention of infectious diseases, cancer, and brain diseases [10,14,15,16,17,18]. The immunogenicity achieved by displaying antigens on VLPs is assumed to be similar to that of the natural virus particle due to specific interactions with pattern recognition receptors of dendritic cells (DCs) [14]. Typical VLPs include bacteriophage P22 (P22), bacteriophage Qβ (Qβ), norovirus capsid, and cowpea mosaic virus (CPMV), among others (Figure 2).

**Figure 3:**
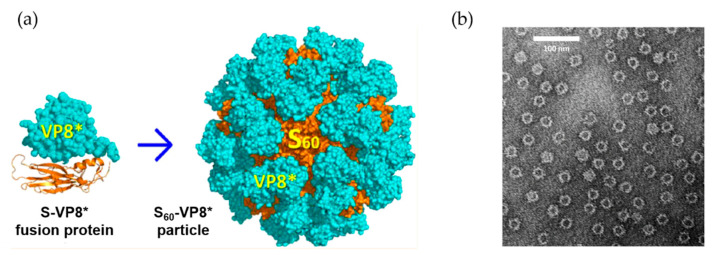
The utilization of norovirus capsid as antigen carrier. (**a**) Scheme for the fusion S domain of norovirus capsid with rotavirus VP8* protein and construction of the assembly structure. (**b**) Electron microscopy images of the S_60_-VP8* particles. Reprinted with permission from [20]. Copyright (2018) American Chemical Society.

Icosahedral and spherical structures of VLPs are effective in display antigens. The norovirus capsid has also been used as a VLP platform for engineering a subunit vaccine [19,20]. In one example, Koho and co-workers modified the capsid by using a simple non-covalent conjugation [19]. Based on the interaction between a surface-exposed polyhistidine-tag and multivalent tris-nitrilotriacetid acid (trisNTA), the capsid surface was decorated with trisNTA-conjugated fluorescent dye molecules and streptavidin-biotin. The potential of this VLP vaccine platform was also demonstrated in delivery of a surface-displayed fluorescent dye into HEK293T cells [19]. Xia et al. employed the norovirus shell (S) domain of the norovirus capsid, which naturally builds the interior shell composed of 60 subunits, known as S_60_. By introducing the rotavirus protein, VP8*, to the C-terminus of the S domain via a linker consisting of four histidine residues (HHHH), a modified version of the S_60_ nanoparticle displaying 60 VP8* proteins was constructed (S_60_-VP8*) (Figure 3) [20]. Mice immunized with the S_60_-VP8* particles exhibit a high IgG response, and the mouse antisera provide strong protection against rotavirus (RV) VP8* binding to glycan ligands. The vaccine nanoparticles also exhibit high neutralizing activity against RV infection in vitro. S_60_ has also been used to display other antigens [20].

There is a promising and highly immunogenic VLP formed by the core protein of hepatitis B virus surface antigen (HBsAg) which is used for the delivery of immunogens from various diseases. For example, a chimeric HBsAg DNA immunogens was developed by replacing the DNA encoding HBsAg-specific cytotoxic T cells (CTL) epitopes by DNA encoding foreign CTL epitopes which showed immunogenic responses [21]. It is also possible to induce simultaneous responses if dual epitopes are combined to the recombinant HBsAg DNA. Hepatitis B core protein (HBcAg) is also a powerful tool in biomolecular engineering. Peyret et al. fused two HBcAg with a flexible GGG linker to form a dimer which can act as a single polypeptide chain [22]. Such a tandemly fused HBsAg dimer can form VLP in bacteria and plants and can enhance the capacity of accommodating foreign proteins at the major insertion region (MIR). Similarly, a HBcAg carrier was developed by the assembly of two polypeptide chains carrying an FG loop from a third domain of immunoglobulin E (IgE) heavy chain which can efficiently form VLPs (SplitCore technology) [23]. Such a method can elicit human IgE antibodies. The stability and assembly of HBc core protein are improved by introducing artificial covalent disulfide bridges and transplanting a rare spike region on the VLP, which has no anti-carrier responses in immunized mice [24]. HBc VLP was recently designed to display dual antigens against melanoma [25] and heterologous antigens from *Neisseria meningitidis* [26].

**Figure 4:**
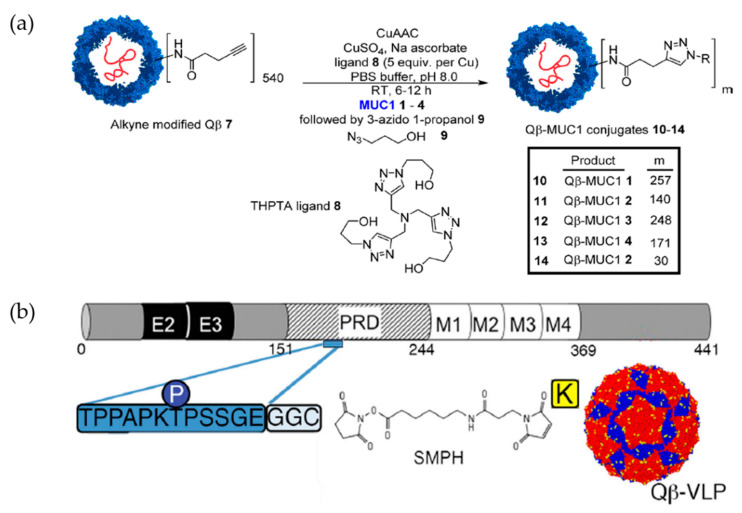
The utilization of Qβ VLP as carrier in vaccine design strategies. (**a**) Scheme for the synthesis of Qβ-MUC1 conjugates for tumor cell targeting. Reprinted with permission from [27]. Copyright (2018) American Chemical Society. (**b**) Conjugation of Qβ VLP to pT181 for Alzheimer’s disease. Abbreviations: E, exon; PRD: protein rich domain; M: microtubule-binding domain. Reproduced with permission from Springer Nature (Creative Commons Attribution 4.0 International License) [16].

Bacteriophage Qβ was used to display a glycopeptide antigen known as MUC-1, which is overexpressed on many cancer cell types [27]. An alkyne-functionalized Qβ VLP was fused with high density of glycosylated and non-glycosylated azide-functionalized MUC-1 peptides (Qβ-MUC1) (Figure 4a). High valency copies of MUC-1 on capsid, Qβ-MUC1 **11**, significantly elicited anti-MUC-1 antibody in comparison to Qβ-MUC1 **14**, which only have 30 copies of MUC-1. Furthermore, Qβ-MUC1 induced generation of killer T cells in vivo and elicits antigen-specific killing of tumor cells [27]. Qβ was also used in development of a vaccine for neurodegenerative diseases [16,28]. Maphis and co-workers introduced Qβ VLP displaying pT181, an engineered tau peptide with phosphorylated threonine 181 and replacement to provide two glycine residues and one cysteine residue (175TPPAPKp**T**PPSSGEGGC190), at the surface-exposed lysines via the bifunctional cross-linker succinimidyl 6-[(beta-maleimidopropionamido) hexanoate] (SMPH) (Figure 4b) [16]. Both non-Tg and rTg4510 mice immunized with pT181- Qβ obtained a long-lasting anti-pT181 antibody response in sera and the brain tissue.

Plant viral nanoparticles are highly ordered and multivalent protein capsids, which are ideal carriers for surface display of antigens [17,29]. Cowpea mosaic virus (CPMV) is a 60-subunit nanoparticle assembled from a 42-kDa (large) coat protein and a 24-kDa (small) coat protein to form a 30-nm assembly (Figure 2). Filamentous potato virus X (PVX) is a flexible 515-nm long and 13-nm wide filament which contains 1270 identical subunits of a 25-kDa capsid protein. These two morphologically distinct plant VLPs were chosen to display human epidermal growth factor receptor 2 (Her2) for humoral response targeting. Morphological contributions were investigated. The antigens were chemically conjugated to VLPs using the heterobifunctional N-hydroxysuccinimide-PEG4- maleimide linker SM-PEG4. These antigens were 70% more effectively internalized via PVX than CPMV, but the CPMV vaccines were more effectively transported into the draining lymph node and had greater uptake by APCs [30].

Some approved vaccines have high humoral immune responses but weak cellular immune responses. Thus, adjuvants are employed to elicit cellular immunity [31]. In recent investigations of P22, Schwarz and co-workers were able to induce infection-protective T cell responses via APC interactions without any adjuvant. The matrix and matrix 2 proteins of respiratory syncytial virus (RSV) as two protein antigens were co-encapsulated into the P22 VLP. The resulting nanoparticle exhibits CD8^+^ and CD4^+^ T cell responses [32].

**Figure 5:**
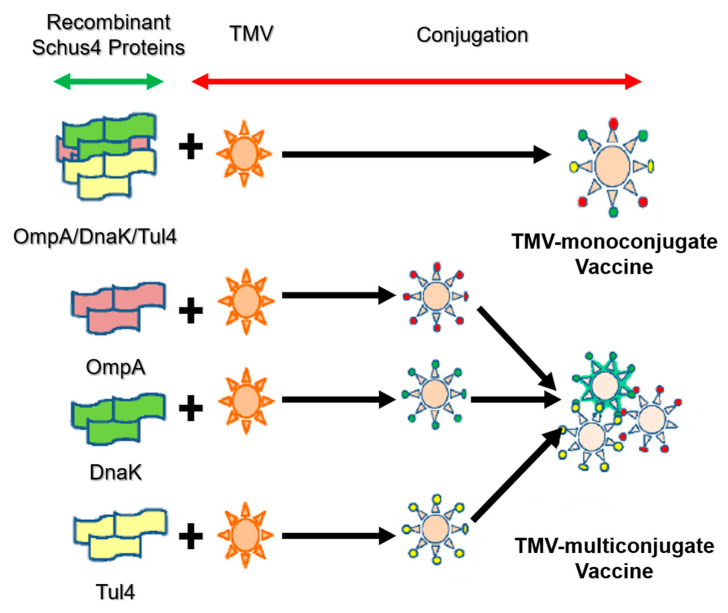
Design strategy of mono- and multi-conjugated vaccine with tobacco mosaic virus (TMV) [34].

In addition to VLPs with spherical and filament morphology, a hollow tubular VLP has been employed as a versatile platform for displaying target antigens and pathogens. One of the most useful platforms is tobacco mosaic virus (TMV), which has been investigated in vaccine development for several diseases such as cancer [33], tularemia [34] and malaria [35]. TMV has been developed as a delivery platform for vaccine applications because it can carry antigen and stimulate cellular responses without requiring additional adjuvants, and because it is not a human pathogen. McCormick and co-workers demonstrated that T-cell mediated immunogenicity and tumor protection could be achieved by chemical conjugation of synthetic peptides to TMV without any additional adjuvant [36]. Banik et al. created a novel multivalent TMV-based delivery carrier by adding a mixture of proteins. The multivalent subunit vaccine contains a mixture of *Francisella tularensis* protective antigens: OmpA-like protein (OmpA), chaperone protein DnaK and lipoprotein Tul4. TMV can be used as a basis for monoconjugate and multiconjugate vaccines to provide highly efficient humoral responses (Figure 5) [34].

## 3. Protein Cages

Similar to VLPs, non-viral protein cages are formed of symmetric, self-assembled nanosized subunits which are capable of repetitive antigen display. Nanoparticles based on prokaryotic and eukaryotic protein cages have been investigated in biocatalysis, nanomaterial synthesis, and molecular transport [10]. A number of protein cages derived from proteins such as ferritin, vault, and E2 have been recently investigated in vaccine development.

### 3.1. Ferritin

**Figure 6:**
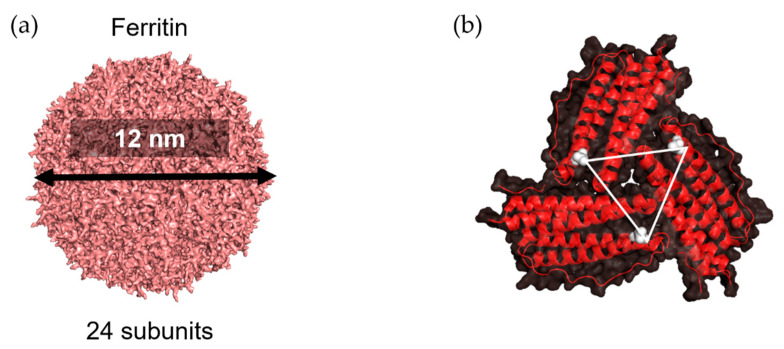
Three-fold axis structure in ferritin. (**a**) Structure of ferritin as 24-subunit assembly (PDB ID: 3BVE using PyMOL). (**b**) A three-fold axis formed by three subunits is shown; the Asp 5 residue in each chain is colored in white; the triangle represents distance among three Asp 5 residues.

Ferritin, a protein cage that serves as an ion storage protein, is found in essentially all living organisms. With a molecular weight of ~450 kDa, ferritin consists of 24 self-assembled subunits that form a spherical cage-like structure [37,38] (Figure 6a). The inner and outer dimensions are 8 and 12 nm, respectively. The ferritin cage reversibly disassembles in acidic (pH 2–3) or basic (pH 10–12) environments [39]. This feature has been investigated with respect to drug loading and preparation of bionanomaterials [38,40,41,42,43,44,45]. Moreover, the unique symmetrical structure of ferritin has been used to display various antigens related to infectious diseases such as influenza [46,47,48], human immunodeficiency virus type-1 (HIV-1) [49,50], hepatitis B virus (HBV) [51], hepatitis C virus [52], and Epstein–Barr virus [53].

The three-fold axis channels of the apo-ferritin cage, a ferritin with a hollow cage, are well suited to provide surfaces to conjugate with antigens whose trimeric structures required stabilization to induce immune responses. Kanekiyo et al. presented influenza virus haemagglutinin (HA) on the surface of 24-subunit apo-ferritin to obtain nanoparticles containing eight trimeric viral spikes which spontaneously assemble because the distance between two Asp 5 residues on the three-fold axis is 28 Å (Figure 6b) [46]. This distance is essentially identical to the distance between the central axes of each HA subunit, HA2 of trimeric HA. This nanoparticle vaccine improves the potency and breadth of influenza virus immunity. The three-fold axis has also been applied in presentation of the trimeric structure of gp120, a receptor-binding protein on the envelope (Env) glycoprotein of HIV-1. A region known as V1V2 of Env, which ranges from 50 to 90 residues in length, was presented in a native-like conformation around the threefold axes on the surface of the apo-ferritin cage [49].

Wei and co-workers also recognized the utility of the three-fold axis channels in a recent study, by employing an apo-ferritin, to encapsulating influenza virus nucleoprotein (NP) antigen peptides within apo-ferritin [48]. NP is expected to play an important role in influenza virus infection via viral genome packaging and replication. An apoferritin–haemagglutinin (HA-AFr) nanoparticle was created with interior NPs. Heating at 50 °C over 45 min causes the AFr cage to expand the spaces between subunits, enabling penetration of NP molecules. Next, the HA antigens were displayed on the surface of apoferritin via chemical conjugation which connect HA and apoferritin via a heterobifunctional PEG cross-linker to form HA-AFr+NP particles. These nanoparticles induce both HA and NP-specific antibodies and provide complete protection in viral challenge. Another dual-targeting nanoparticle vaccine using the ferritin nanoparticle has recently been developed against hepatitis B virus (HBV) [51] via a “plug-and-display” system known as SpyTag-SpyCatcher, a spontaneous isopeptide bond formation tool [54]. This nanoparticle vaccine has high potential for specific myeloid cell targeting when preS1 is successfully delivered to T follicular helper cell activator (SIGNR1+ dendritic cells) and B cell activator (lymphatic sinus-associated SIGNR1+ macrophages) [51].

### 3.2. Other Self-Sssembled Protein Structures 

In addition to the widely used VLPs and ferritins, there are examples of self-assembled structures in vaccine development which include an oligomerizing domain known as IMX313, which is based on chicken complement inhibitor C4b-binding protein with seven subunits. Li et al. have fused Pfs25, the leading malaria transmission-blocking target to IMX313 to form heptameric protein nanoparticles [55]. By comparison, monomeric Pfs25 provides less immunogenic and transmission-reducing activity than the Pfs25- IMX313 nanoparticle vaccine. To increase the amount of antigen conjugated to the self-assembled proteins as an alternative to the 8 or 24 units provided by ferritin or the 7 units provided by IMX313, several researchers have employed lumazine synthase for HIV vaccination [56,57]. Lumazine synthase can display 60 units of gp120, an envelope glycoprotein of HIV-1.

Allen and Perham isolated E2 protein from the E2 core domain (dihydrolipoamide acetyltransferase) of the pyruvate dehydrogenase complex derived from *Bacillus stearothermophilus* [58]. The E2 cage has a diameter of 25 nm and contains 60 subunits forming a pentagonal dodecahedral cage with icosahedral symmetry. Multiple foreign peptides and proteins can be linked to surface of this protein cage [59]. Caivano et al. decorated the N-terminus of E2 protein derived from *Geobacillus stearothermophilus* with HIV-1 Gag(p17) protein [60]. The Gag(p17)-E2 60-mer particles stimulate a strong and well-preserved antibody response in immunized mice. Despite the absence of IFNγ-producing CD4+ T cells, the nanoparticle vaccine can evoke production and activity of antibody- and antigen-specific cytotoxic T cells (CTL). These results indicate that the E2 cage can induce strong humoral and cellular immune responses. The E2 cage has also been utilized to deliver a combination of two human cancer-testis peptide epitopes, NY-ESO-1 epitope (SLLMWITQV) and MAGE-A3 epitope (FLWGPRALV) with an adjuvant to obtain significantly enhanced cellular immune responses [61].

**Figure 7:**
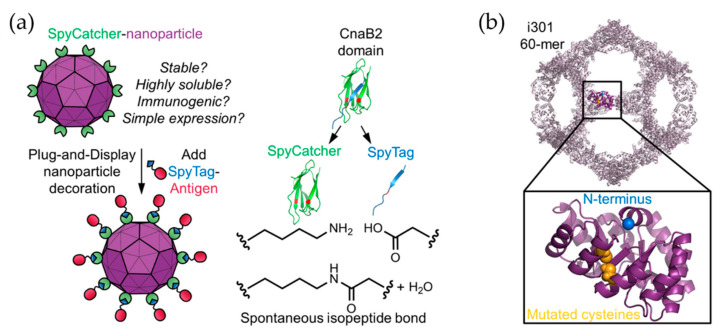
i301 as a platform for antigen display. (**a**) An illustration of a Plug-and-Display system using SpyCatcher-i301 and a SpyTag-antigen. (**b**) Modification of the i301 carrier, C76 and C100 in i301 (yellow), which were mutated to alanine to produce the mi3 nanoparticle platform. Reprinted with permission from [70]. Copyright (2018) American Chemical Society.

**Figure 8:**
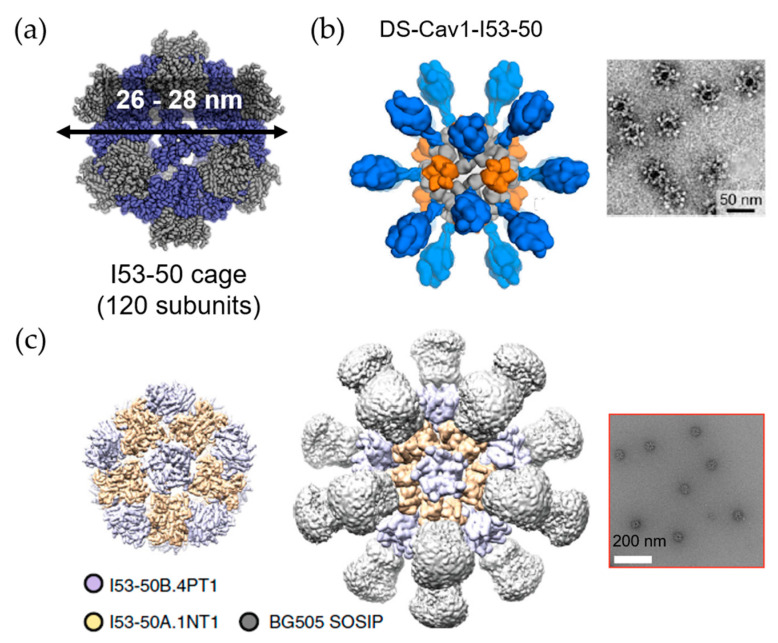
The utilization of I53-50 cage in vaccine design. (**a**) Design of I53-50 cage composed with 120 subunits of two component proteins. Modified from [71]. Reprinted with permission from American Association for the Advancement of Science (AAAS). (**b**) The utilization of I53-50 as a carrier for respiratory syncytial virus antigen (DS-Cav1) conjugation; and (**c**) HIV-1 antigen (BG505 SOSIP) conjugation with negative stain electron microscopy (EM) images on the right side, respectively. Reproduced with modification from [72] and [73].

Vault proteins exist in essentially all eukaryotic cells. Recombinant vault cages consist of 96 subunits from the major vault protein (MVP) and have dimensions of approximately 70 nm × 40 nm × 40 nm. These hollow barrel shaped cage-like proteins are assembled from 13-MDa ribonucleoproteins [62,63]. By decorating the N-terminus of MVP with different tags, the recombinant vault proteins were used to target specific cells via cell surface receptors [64]. A membrane lytic peptide derived from adenovirus protein VI (pVI) was fused to the N-terminus of the major vault protein to form recombinant vault proteins known as pVI-vaults. Higher levels of transfection efficiency were achieved with fewer vault cages [65]. Kelly and coworkers engineered vault proteins to contain major outer membrane protein (MOMP), an enriched T- and B-cell epitopes antigen. By fusing MOMP with minimal interaction protein, MOMP is allowed to pack within the vault proteins. Vaccines based on this platform stimulate adaptive immune responses and protective immunity [66]. Similarly, ovalbumin (OVA) was encapsulated within vault cages to produce vaccines which efficiently elicit robust CD8+, CD4+ memory T cell responses and OVA-specific antibodies. These results indicate that the utilization of vault cages as subunit vaccine carriers can generate both cellular and humoral immune responses [67]. Another study from Kelly’s group has promoted the protective capacity of vault cage vaccines in the absence of additional adjuvants when a peptide from polymorphic membrane protein G-1 (PmpG) of *Chlamydia muridarum* is encapsulated within the vault cages [68]. Despite the versatility of natural protein cages, misfolding may occur during expression of a mixture of protein cage and components to be encapsulated. Thus, computationally designed artificial nanoparticles with cage structures have been investigated. A platform known as i301, formed of 60 subunits with porous dodecahedral architecture, was designed by Hsia et al. [69]. Brunn and co-workers investigated a SpyCatcher fusion of mi3, an i301 mutant, and an antigen of interest linked to the SpyTag peptide (Figure 7) [70]. The resulting computationally designed cage vaccine generates a high avidity immunity, which is comparable to immunity produced by phage-derived VLPs. Another useful computationally designed platform is I53-50 [71]. I53-50 is an assembly of two different component proteins: 20 trimeric “A” components and 12 pentameric “B” components to provides a total of 120 subunits aligned along five-fold and three-fold icosahedral symmetry axes (Figure 8a) [71]. Two distinct oligomeric protein components can be expressed and purified separately and assembled efficiently into highly ordered, homogenous icosahedral particles with diameters ranging from 24 to 40 nm. This platform with a highly repetitive surface has recently been employed in vaccinations against infectious diseases such as and respiratory syncytial virus (Figure 8b) [72] and HIV-1 (Figure 8c) [73]. These nanoparticle vaccines generate monodisperse immunogens with high efficiency [72], and are particularly efficacious as priming immunogens which improve the quality of the antibody response [73].

## 4. De Novo Peptides

De novo peptides are not based on any natural self-assembled proteins. Design of de novo peptides includes construction of secondary structures promoting self-assembly via hydrophobic or electrostatic interactions. An advantage of de novo peptide design for vaccine applications is to avoid anti-carrier responses [74]. Complications remain in engineering efforts and design strategies.

De novo peptides form unstructured hydrophobic assemblies such as amphiphilic assemblies or aggregates. The amphiphilic micelles have potent self-adjuvanting vaccine properties. The micelle size and charge have an influence on bioactivities such as lymph node accumulation, cell uptake ability, and immunogenicity [75].

Despite significant achievements in design to date, such unstructured assemblies have resulted in fewer applications than structured and ordered assemblies including β-sheets and α-helices.

### 4.1. β-Sheet Fiber

Two common β-sheet domains, Q11 (QQKFQFQFEQQ) and KFE8 (FKFEFKFE), have been employed in vaccine development. The engineered Q11 domain can assemble into nanofibers following conjugation with a peptide antigen at its N-terminus via a short linker. The self-assembled nanofibers are stacked assemblies displaying target antigen outward. This method has been applied to display epitopes from infectious disease viruses. The malarial peptide epitope from *Plasmodium falciparum* circumsporozoite (CS) protein, (NANP)_3_, was linked to form (NANP)_3_-Q11 [76]. In another example, an epitope from influenza acid polymerase (PA) (PA224–233, SSLENFRAYV) was linked to create PA224–233-Q11 fibers [77]. One of the most outstanding advantages of the Q11 domain is its capacity to conjugate to different kinds of epitopes. The self-assembled Q11 nanofibers form easily by mixing, and the ratio of epitopes displayed on Q11 fibers can be controlled via intermixed and separately assembled methodologies in a concentration-dependent manner [78]. For instance, there are reports of fibrils displaying both B cell epitope and T cell epitope on the outer surface of Q11 fibers [79,80]. Despite the absence of additional adjuvants, these materials induce antibody responses against autologous TNF (tumor necrosis factor) in mice. The strength of the anti-TNF antibody response is adjustable via the epitope content in the nanofibers [80]. Moreover, surface charges of epitopes correlate with immune responses via uptake of APCs. Wen et al. investigated the correlation via a range of physicochemical properties of fibrillized peptide biomaterials including negatively and positively charged peptides. The negative surface charge prevents the uptake of APCs and display of epitope peptide in the major histocompatibility class II molecules of APCs, which leads to prevention of T cell and antibody responses. However, the positive surface charge increases uptake of fibrillized peptides by APCs [81].

### 4.2. α-Helix

**Figure 9:**
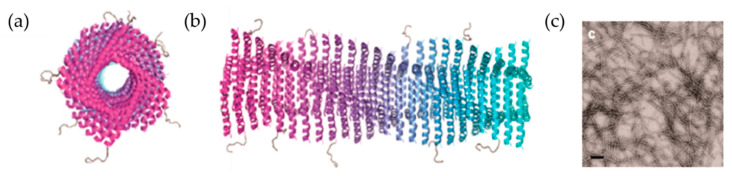
Self-assembled structures of Coil29 in α-helical nanofibers. (**a**,**b**) Axial view and side view schematics of PEPvIII epitopes displaying outward of Coil29 fibers at the N-terminus. (**c**) Negative stain TEM image of Coil29 nanofibers with a scale bar of 100 nm. Reproduced with modification from [84]. Copyright (2017) American Chemical Society.

Although the β-sheet is a useful platform for presentation of multiple types of epitope-bearing peptides, the resulting structures have some shortcomings with respect to control of assembly and disassembly kinetics; insufficient structural precision in topology of individual β-strands, and control of lengths and interactions between nanofibers [82,83]. A self-assembly peptide known as Coil29 (QARILEADAEILRAYAR- ILEAHAEILRAQ), has a predominant α-helix structure and a long axis directed toward perpendicular α-helical nanofibers (Figure 9). The N-terminus extension is directed outward toward the surface of the nanofiber. This provides an advantage for Coil29 in conjugation to cargo proteins, indicating that this structure should be investigated as a potential candidate for a vaccine platform [84]. When Coil 29 is decorated with various epitopes of B cells and T cells such as a cancer B-cell epitope from the epidermal growth factor receptor class III variant (EGFRvIII), a universal CD4+ T-cell epitope PADRE, and a model CD8+ T-cell epitope SIINFEKL, the nanofibers induce a strong antibody response and are readily incorporated into antigen presenting cells. Immunized mice demonstrate CD4+ T-cell and CD8+ T-cell responses without supplemental adjuvants [84]. In a similar manner, self-adjuvanted self-assembling protein nanoparticles (SAPNs) were generated by exhibition of the two conserved influenza antigens M2e and Helix C in their native oligomerization states with incorporation of the TLR5 agonist flagellin into the SAPNs [85]. Chickens vaccinated with the self-adjuvanted SAPNs generate high levels of antibodies with high cross-neutralizing activity in comparison with a commercial inactivated virus vaccine. These results indicate the potential for development of a universal influenza vaccine based on SAPNs.

## 5. Natural Building Blocks

Other than the protein assemblies described above, supramolecular proteins consisting of different protein assemblies are also attracting attention as candidates for the development of vaccine platforms. Several bacteriophages have been investigated [86]. Bacteriophage T4, one of the best known assemblies of supramolecular proteins, has been studied extensively in terms of its function as a molecular machine [87]. The component proteins of bacteriophage T4 have also been investigated in context of developing vaccine carriers.

Bacteriophage T4 (T4) is constructed from more than 40 different protein building blocks forming a head structure, a tail structure, and fibers attached to the distal end of the baseplate [88]. The fibers recognize the host cell surface and then the entire supramolecular structure of T4 promotes the motion required to inject the DNA into the cell [88]. The tail can serve as a constricting tube for delivering the T4 DNA from the head into the host cell. Its unique architecture provides T4 bacteriophage with useful features for developing a powerful vaccine platform. For instance, the head provides a high repetitive surface with thousand protein molecules; the foldon domain from the fiber is commonly used to stabilize trimeric proteins. Protein engineering efforts have focused on the head and on the foldon domain for vaccine carrier applications (Table 1).

**Figure 10:**
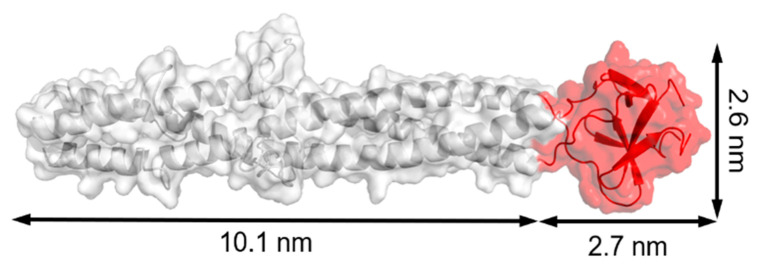
Model structure of fibritin E (from Glu368 to Ala486 of fibritin) from PDB ID: 1AA0. The foldon domain at the C-terminus is presented in red with dimensions of 2.7 × 2.6 nm. The structure was rebuilt by PyMOL.

The building block of T4 has been used to stabilize an assembled subunit. There is a 53-nm long fiber encoded by the *wac* gene, which is known as fibritin [105]. Its trimer structure is stabilized by the foldon domain with 30 residues at the C-terminus (Figure 10). Trimer structures of collagen, the gp26 fiber from bacteriophage P22, and the gp5 needle protein from bacteriophage T4 were stabilized by fusing them to the foldon domain [106,107,108]. The function of foldon has been applied to construct subunit vaccines including target trimer subunits as antigens of HIV-1, RSV, influenza virus, middle east respiratory syndrome coronavirus (MERS-CoV), and severe acute respiratory syndrome coronavirus 2 (SARS-CoV-2).

Foldon was used to stabilize the protein trimer of envelope glycoprotein in HIV-1 [89]. It was reported that soluble gp140, a glycoprotein composed of un-cleaved external domains of gp120 and gp41, form a trimer fused by the GCN4 motif [109]. When foldon was used as the C-terminal domain of gp140 instead of GCN4, the solubility and thermal stability of the gp140 trimer were improved while maintaining the antigenicity of gp120.

The highly pathogenic avian influenza (HPAI) H5N1 virus can infect humans with a high mortality rate. Influenza virus hemagglutinin (HA) is known as a target for the subunit vaccines. HA is a homo-trimeric membrane glycoprotein on the surface of the virus. The HA monomer is first synthesized as a precursor and then the host protease cleaves the monomer into two fragments known as HA1 and HA2 [97]. Du and co-workers prepared HA1 -fused human IgG1 Fc (Fc) and foldon to maintain the trimer structure and immunogenicity because Fc is known to significantly improve the immunogenicity of subunit vaccines [97]. The fusion protein has adjuvant properties in inducing the humoral immune response and a strong antibody response via the mucosal pathway. This result showed that proteins capable of maintaining the natural trimer structure of HA could elicit a stronger immune response than monomers.

RSV is a severe disease for children. Palivizumab (Synagis) is a therapeutic antibody against RSV-fused (F) glycoproteins which prevents the disease by passive prophylaxis. It was found that the main target of RSV neutralizing antibodies induced by natural infection is the prefusion conformation of RSV F. To enhance induction of potent antibodies, McLellan designed a soluble mutant of RSV F with a stable exposed antigen site Ø [99]. Foldon was added to the C-terminus of the RSV F external domain for structural analysis. The stable presentation of the antigenic site Ø was retained with the C-terminal trimerized domain and other modifications, such as introduced disulfide bonds and hydrophobic interactions. Over 100 fusion glycoprotein variants were designed, evaluated for antibody reactivity, and analyzed for their ability to elicit a protective response. This structural vaccinology approach provides an immunogen that elicits an improved protective response relative to the post-fusion form of fused glycoprotein in one of the RSV vaccine candidates.

MERS-CoV was first identified in 2012. The native spikes (S) of MERS-CoV are enveloped glycoproteins presented as viral surface trimers which are cleaved into S1 and S2 subunits by host cell proteases during viral infection. S1 is involved in the process of binding of MERS-CoV to host cells that express the viral receptor dipeptidyl peptidase 4 (DPP4) because it contains the receptor-binding domain (RBD). A recombinant trimer RBD protein was synthesized as an antigen by fusing the RBD sequence in S1 of MERS-CoV with foldon [101]. The trimer recognizes RBD-specific antibodies and binds strongly to monoclonal antibodies targeting MERS-CoV RBD-specific conformational epitopes and the MERS-CoV receptor DPP4. The trimer can elicit highly effective IgG antibodies via Th2-based IgG1 and Th1-based IgG2 antibody responses. These results show that the MERS-CoV RBD trimer maintains original immune functions by stabilization via foldon. In addition, construction of a recombinant trimer of RBD for SARS-CoV-2S1 by fusing SARS-CoV-2S1 with foldon has recently been reported [102].

In addition to foldon domain, the head of T4 is capable of efficiently presenting subunits of target viruses as antigens. The head structure includes more than 2000 protein molecules and has a molecular mass of 82 MDa [110]. The characteristic structure is a prolate icosahedron with one unique portal vertex to which the tail is connected. The head has a hexagonal surface lattice constructed of hexamers of gene product (gp)23* and pentamers of gp24*. The outer surface is formed of highly antigenic outer capsid protein (hoc) and small outer capsid protein (soc) [110]. Jiang et al. have developed a phage display system that enables fusion of foreign proteins using a unique DNA cloning site at the 5’ end of hoc or soc. A 36-amino acid PorA peptide from *Neisseria meningitidis* was fused to the N-terminus of hoc or soc. PorA-hoc and PorA-soc fusion proteins were presented on the head surface by enzyme-bound immunoadsorption while maintaining reactive activity with a particular monoclonal antibody [111].

**Figure 11:**
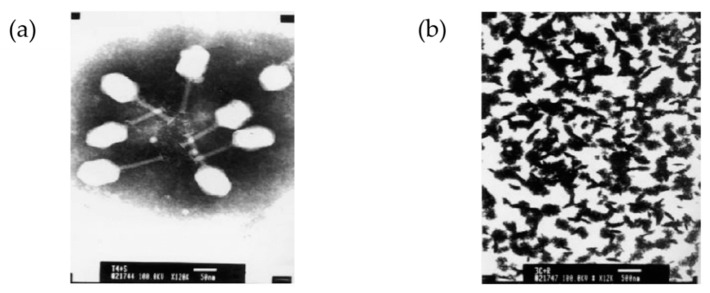
The head of bacteriophage T4 as a pathogenic display system. (**a**) Negatively stained image of the recombinant T4-3C phage particles (scale bar: 50 nm, magnification 12kx); (**b**) aggregation–precipitation of phages due to the addition of pig anti- foot-and-mouth disease (FMDV)-O antibody into the mixture of E. coli BL21 (DE3) infected phage T4-P1 and recombinant T4-3C phage particles (scale bar: 500 nm, magnification 12kx). Reprinted from [103], copyright (2008), with permission from Elsevier.

A T4 head surface gene-protein display system (T4-S-GPDS) was used to create a foot-and-mouth disease virus (FMDV) vaccine in FMDV O serotype (FMDV/O) [103]. FMDV capsid precursor polyprotein (P1, 755 aa) or proteinase 3C (213 aa) was genetically fused into soc, which is exposed on the outer shell of the head but not essential for stabilizing the head. Mixing the T4 mutants with porcine anti-CD-10 IgG resulted in phage immuno-precipitation (Figure 11). This indicates that P1 displayed on the surface of the T4 head can also be treated with the 3C proteinase displayed on T4. This immunogen is recognized by an FMDV/O-specific antibody. When mice were immunized via oral administration or injection with a mixture of T4_P1 and T4_3C particles without the addition of adjuvants, they were protected from the disease. This investigation of T4-S-GPDS has shown that various types and subtypes of unstructured viral proteins of FMDV can be labeled on T4 as reporter antigens in development of diagnostic methods.

**Figure 12:**
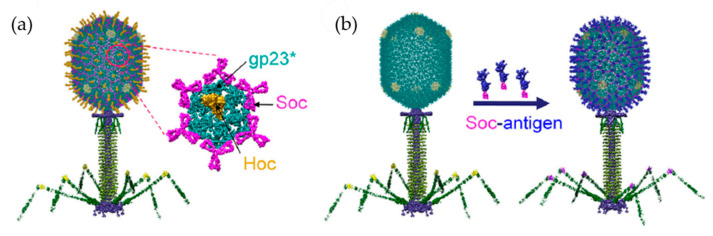
Scheme of the bacteriophage T4 nanoparticle platform and assembly design. (**a**) Structural model of phage T4 with the major capsid protein gp23* (cyan) (the asterisk shows that it is the cleaved form) (930 copies), soc (magenta) (870 copies), and hoc (yellow) (155 copies). (**b**) In vitro assembly of Soc-fused antigen (blue) molecules on hoc_ soc_ T4 phage capsid. Reprinted from [104].

A multivalent vaccine against both anthrax and plague was developed using a modification of the T4 head [104]. It is known that both the N-terminus and C-terminus of soc are exposed on the surface of the T4 head. Target antigens were fused to both ends, and dual recombinant proteins can be efficiently displayed. Anthrax PA (83 kDa) and plague F1mutV (56 kDa) were fused to soc at the N-terminus and C-terminus, respectively (Figure 12). The modified soc proteins were assembled in vitro on the T4 head. The high-affinity between soc and the head provides tight anchoring of the antigens in a symmetrical arrangement in the icosahedral lattice. The dual valent vaccine elicits high titers of antigen-specific antibodies against both anthrax and plague antigens. In addition, the double anthrax plague vaccine elicits a strong immune response against both antigens in the absence of adjuvant.

Zhu and coworkers developed a hybrid viral vector in which the T4 head is ligated to adeno-associated virus (AAV) via an avidin–biotin interaction with soc or hoc. Creating the largest hybrid vector capable of delivering exogenous lengths of DNA up to 170 kb and up to 1025 protein molecules into human cells [112]. The T4 head encapsulating luciferase DNA (~6.2 kb), displaying E. coli-derived β-galactosidase (116 kDa) ~250 copies via soc and GFP-packaged AAV via hoc, were delivered to HEK293 cells. The expected fluorescence of luciferase, GFP and activity of β-galactosidase were confirmed. The hybrid vaccine, which incorporates the HA DNA and displays the F1mutV antigen from *Y. pestis*, induces an immune response against the influenza virus and the *Y. pestis* pathogen.

## 6. Perspectives

Subunit vaccines are safe, simple, and capable of rapidly inducing immunogenicity but are fragile with respect to immunological memory induction. Engineered subunit vaccines including biomaterial carriers for antigen display have helped in addressing this challenge with successes in improvement of humoral and cellular immune responses, along with their modifiable structures. In this review, we have discussed a variety of protein assembly structures as building blocks which have provided promising results in subunit vaccine development. Although organic or polymeric nanoparticles can deliver pathogenic fragments, there are several advantages of using protein assemblies: (1) self-assembly enables retention of multiple pathogenic units within a single assembly structure keeping the original activity of the assembly; (2) their sizes enhance lymph node trafficking and delivery of pathogenic portions into the cell to increase the efficacy of the vaccine; and (3) they can prevent the aggregation of the pathogenic portions and can be re-designed to alter the orientation of the pathogenic portions. Protein assemblies can include viral-like particles and non-viral self-assembled proteins including ferritin, E2 cage, vault cage, computationally derived cage-like structures, de novo peptides, and foldon from bacteriophage T4, among others. Therefore, protein assembly structures are considered as useful platforms for developing subunit-based vaccines. Despite the promises of such protein-based subunit vaccines with respect to safety, it remains challenging to establish efficacy and to efficiently select pathogen portions to be fused with the subunits to provide optimal activity.

VLPs, which resemble viral capsid structures and do not contain genetic materials, have advantages such as the ability to display monovalent or multivalent target antigens. VLPs can be engineered to precisely target immune cells (DCs for T-cell activation) and antigen presentation of VLPs can be improved by using linkers with different properties [14]. These structures are mimicked from viral structures. However, development of efficient design strategies remains challenging with respect to selection of VLP candidates according to the type of immune response for universal vaccine applications [14,86]. On the other hand, non-viral proteins include natural and artificial peptides and protein assemblies representing a library of platforms with precise design and controllable structures. For instance, display of epitopes on Q11 fibrils is adjustable and protein cages can be modified to exhibit pathogenic portions on their surfaces or encapsulate them within hollow cages. Together with the advantages of both VLPs and non-VLPs, the head and foldon domain of bacteriophage T4 provide additional natural building blocks. Bacteriophage T4 is highly stable and can be efficiently manufactured, making it a promising candidate as a universal vaccine delivery vehicle for prevention of infectious diseases [86].

In an effective response to a pandemic, rapid production of an effective and safe vaccine is essential. Subunit vaccines based on protein assemblies are useful in such efforts because their original assembly structures and properties such as cellular delivery characteristics and tolerability are known. Other than the natural building blocks or related assemblies, computational and structural biology can provide additional guidance in rapid design, development and screening of a large number of protein assembly candidates. Additionally, screening linkers may help present target antigens in the APCs more effectively. We are hopeful that this review will provide useful background information and guidance in computational design efforts to produce new protein assemblies based on natural building blocks. Artificial platforms constructed by rational design from viral proteins providing stable and functional building blocks, are promising candidates for the development of vaccines against infectious disease.

## Figures and Tables

**Table 1:** Bacteriophage T4 building blocks as carriers for vaccines against infectious diseases.

Carriers	Target Disease(s)	Antigen(s)	References
fibritin	HIV-1	Uncleaved, soluble gp140 glycoproteins	[89]
fibritin	HIV-1	a noncleavable gp140 envelope protein	[90]
foldon	HIV-1	gp140	[91,92,93]
foldon	influenza	HA	[94,95]
foldon	influenza	HA stem domain	[96]
foldon	influenza	HA1	[97,98]
foldon	RSV	RSV fusion (F) glycoprotein	[99,100]
foldon	MERS-CoV	Receptor-binding domain (RBD)	[101]
foldon	MERS-CoV	MERS-CoV-S1, SARS-CoV-2-S1	[102]
soc	Foot-and-mouth disease (FMD)	P1, proteinase 3C	[103]
soc	Anthrax, plague	Anthrax PA, plague F1mutV	[104]

Abbreviations: HA, haemagglutinin; RSV, respiratory syncytial virus; MERS-CoV, middle east respiratory syndrome coronavirus; PA, protective antigen.

## Data Availability

No datasets were generated or analyzed in this study.

## References

[1] Luo Q., Hou C., Bai Y., Wang R., Liu J. (2016). Protein Assembly: Versatile Approaches to Construct Highly Ordered Nanostructures. Chem. Rev..

[2] Pieters B.J.G.E., Van Eldijk M.B., Nolte R.J.M., Mecinović J. (2016). Natural supramolecular protein assemblies. Chem. Soc. Rev..

[3] Ulijn R.V., Smith A.M. (2008). Designing peptide based nanomaterials. Chem. Soc. Rev..

[4] Smith D.M., Simon J.K., Baker J.R. (2013). Applications of nanotechnology for immunology. Nat. Rev. Immunol..

[5] Punt J., Stranford S.A., Jones P.P., Owen J.A., Schultz L., Champion E., Frost E.P., Compton J., Davidson K. (2019). Infectious Diseases and Vaccines. Kuby Immunology.

[6] Skwarczynski M., Toth I. (2016). Peptide-based synthetic vaccines. Chem. Sci..

[7] Sompayrac L.M. (2019). How the immune system works.

[8] Moyer T.J., Zmolek A.C., Irvine D.J. (2016). Beyond antigens and adjuvants: Formulating future vaccines. J. Clin. Investig..

[9] Bachmann M.F., Jennings G.T. (2010). Vaccine delivery: A matter of size, geometry, kinetics and molecular patterns. Nat. Rev. Immunol..

[10] Tsoras A.N., Champion J.A. (2019). Protein and Peptide Biomaterials for Engineered Subunit Vaccines and Immunotherapeutic Applications. Annu. Rev. Chem. Biomol. Eng..

[11] Chattopadhyay S., Chen J.-Y., Chen H.-W., Hu C.-M.J. (2017). Nanoparticle Vaccines Adopting Virus-like Features for Enhanced Immune Potentiation. Nanotheranostics.

[12] Ahsan F., Rivas I.P., Khan M.A., Suárez A.I.T. (2002). Targeting to macrophages: Role of physicochemical properties of particulate carriers—liposomes and microspheres—on the phagocytosis by macrophages. J. Control. Release.

[13] Hardy C.L., LeMasurier J.S., Mohamud R., Yao J., Xiang S.D., Rolland J.M., O’Hehir R.E., Plebanski M. (2013). Differential Uptake of Nanoparticles and Microparticles by Pulmonary APC Subsets Induces Discrete Immunological Imprints. J. Immunol..

[14] Zepeda-Cervantes J., Ramírez-Jarquín J.O., Vaca L. (2020). Interaction Between Virus-Like Particles (VLPs) and Pattern Recognition Receptors (PRRs) From Dendritic Cells (DCs): Toward Better Engineering of VLPs. Front. Immunol..

[15] Feng G., Zheng C., Hui J. (2018). Early Aβ-HBc virus-like particles immunization had better effects on preventing the deficit of learning and memory abilities and reducing cerebral Aβ load in PDAPP mice. Vaccine.

[16] Maphis N.M., Peabody J., Crossey E., Jiang S., Ahmad F.A.J., Alvarez M., Mansoor S.K., Yaney A., Yang Y., Sillerud L.O. (2019). Qß Virus-like particle-based vaccine induces robust immunity and protects against tauopathy. NPJ Vaccines.

[17] Steinmetz N.F. (2010). Viral nanoparticles as platforms for next-generation therapeutics and imaging devices. Nanomed. Nanotechnol. Biol. Med..

[18] Yildiz I., Shukla S., Steinmetz N.F. (2011). Applications of viral nanoparticles in medicine. Curr. Opin. Biotechnol..

[19] Koho T., Ihalainen T.O., Stark M., Uusi-Kerttula H., Wieneke R., Rahikainen R., Blazevic V., Marjomäki V., Tampé R., Kulomaa M.S. (2015). His-tagged norovirus-like particles: A versatile platform for cellular delivery and surface display. Eur. J. Pharm. Biopharm..

[20] Xia M., Huang P., Sun C., Han L., Vago F.S., Li K., Zhong W., Jiang W., Klassen J.S., Jiang X. (2018). Bioengineered Norovirus S60Nanoparticles as a Multifunctional Vaccine Platform. ACS Nano.

[21] Woo W.-P., Doan T., Herd K.A., Netter H.-J., Tindle R.W. (2006). Hepatitis B Surface Antigen Vector Delivers Protective Cytotoxic T-Lymphocyte Responses to Disease-Relevant Foreign Epitopes. J. Virol..

[22] Peyret H., Gehin A., Thuenemann E.C., Blond D., El Turabi A., Beales L., Clarke D., Gilbert R.J.C., Fry E.E., Stuart D.I. (2015). Tandem Fusion of Hepatitis B Core Antigen Allows Assembly of Virus-Like Particles in Bacteria and Plants with Enhanced Capacity to Accommodate Foreign Proteins. PLoS ONE.

[23] Baltabekova A.Z., Shagyrova Z.S., Kamzina A.S., Voykov M., Zhiyenbay Y., Ramanculov E.M., Shustov A.V. (2015). SplitCore Technology Allows Efficient Production of Virus-Like Particles Presenting a Receptor-Contacting Epitope of Human IgE. Mol. Biotechnol..

[24] Lu Y., Chan W., Ko B.Y., VanLang C.C., Swartz J.R. (2015). Assessing sequence plasticity of a virus-like nanoparticle by evolution toward a versatile scaffold for vaccines and drug delivery. Proc. Natl. Acad. Sci. USA.

[25] Cheng K., Du T., Li Y., Qi Y., Min H., Wang Y., Zhang Q., Wang C., Zhou Y., Li L. (2020). Dual-Antigen-Loaded Hepatitis B Virus Core Antigen Virus-like Particles Stimulate Efficient Immunotherapy Against Melanoma. ACS Appl. Mater. Interfaces.

[26] Aston-Deaville S., Carlsson E., Saleem M., Thistlethwaite A., Chan H., Maharjan S., Facchetti A., Feavers I.M., Siebert C.A., Collins R.F. (2020). An assessment of the use of Hepatitis B Virus core protein virus-like particles to display heterologous antigens from Neisseria meningitidis. Vaccine.

[27] Yin Z., Wu X., Kaczanowska K., Sungsuwan S., Comellas-Aragonès M., Pett C., Yu J., Baniel C., Westerlind U., Finn M. (2018). Antitumor Humoral and T Cell Responses by Mucin-1 Conjugates of Bacteriophage Qβ in Wild-type Mice. ACS Chem. Biol..

[28] Chackerian B., Rangel M., Hunter Z., Peabody D.S. (2006). Virus and virus-like particle-based immunogens for Alzheimer’s disease induce antibody responses against amyloid-β without concomitant T cell responses. Vaccine.

[29] Shukla S., Wen A.M., Ayat N.R., Commandeur U., Gopalkrishnan R., Broome A.-M., Lozada K.W., Keri R.A., Steinmetz N.F. (2014). Biodistribution and clearance of a filamentous plant virus in healthy and tumor-bearing mice. Nanomedicine.

[30] Shukla S., Myers J.T., Woods S.E., Gong X., Czapar A.E., Commandeur U., Huang A.Y., Levine A.D., Steinmetz N.F. (2017). Plant viral nanoparticles-based HER2 vaccine: Immune response influenced by differential transport, localization and cellular interactions of particulate carriers. Biomater..

[31] Perrie Y., Mohammed A.R., Kirby D.J., McNeil S.E., Bramwell V.W. (2008). Vaccine adjuvant systems: Enhancing the efficacy of sub-unit protein antigens. Int. J. Pharm..

[32] Schwarz B., Morabito K.M., Ruckwardt T.J., Patterson D.P., Avera J., Miettinen H.M., Graham B.S., Douglas T. (2016). Viruslike Particles Encapsidating Respiratory Syncytial Virus M and M2 Proteins Induce Robust T Cell Responses. ACS Biomater. Sci. Eng..

[33] Yin Z., Nguyen H.G., Chowdhury S., Bentley P., Bruckman M.A., Miermont A., Gildersleeve J.C., Wang Q., Huang X. (2012). Tobacco Mosaic Virus as a New Carrier for Tumor Associated Carbohydrate Antigens. Bioconjugate Chem..

[34] Banik S., Mansour A.A., Suresh R.V., Wykoff-Clary S., Malik M., McCormick A.A., Bakshi C.S. (2015). Development of a Multivalent Subunit Vaccine against Tularemia Using Tobacco Mosaic Virus (TMV) Based Delivery System. PLoS ONE.

[35] Langowski M.D., Khan F.A., Bitzer A.A., Genito C.J., Schrader A.J., Martin M.L., Soto K., Zou X., Hadiwidjojo S., Beck Z. (2020). Optimization of a Plasmodium falciparum circumsporozoite protein repeat vaccine using the tobacco mosaic virus platform. Proc. Natl. Acad. Sci. USA.

[36] McCormick A.A., Corbo T.A., Wykoff-Clary S., Palmer K.E., Pogue G.P. (2006). Chemical Conjugate TMV−Peptide Bivalent Fusion Vaccines Improve Cellular Immunity and Tumor Protection. Bioconjugate Chem..

[37] Ford G.C., Harrison P.M., Rice D.W., Smith J.M.A., Treffry A., White J.L., Yariv J. (1984). Ferritin: Design and formation of an iron-storage molecule. Philos. Trans. R. Soc. B: Biol. Sci..

[38] Truffi M., Fiandra L., Sorrentino L., Monieri M., Corsi F., Mazzucchelli S. (2016). Ferritin nanocages: A biological platform for drug delivery, imaging and theranostics in cancer. Pharmacol. Res..

[39] Kim M., Rho Y., Jin K.S., Ahn B., Jung S., Kim H., Ree M. (2011). pH-dependent structures of ferritin and apoferritin in solution: Disassembly and reassembly. Biomacromolecules.

[40] Falvo E., Tremante E., Arcovito A., Papi M., Elad N., Boffi A., Morea V., Conti G., Toffoli G., Fracasso G. (2016). Improved Doxorubicin Encapsulation and Pharmacokinetics of Ferritin–Fusion Protein Nanocarriers Bearing Proline, Serine, and Alanine Elements. Biomacromolecules.

[41] Han J.-A., Kang Y.J., Shin C., Ra J.-S., Shin H.-H., Hong S.Y., Do Y., Kang S. (2014). Ferritin protein cage nanoparticles as versatile antigen delivery nanoplatforms for dendritic cell (DC)-based vaccine development. Nanomed. Nanotechnol. Biol. Med..

[42] Kim S., Jeon J.-O., Jun E., Jee J., Jung H.-K., Lee B.-H., Kim I.-S., Kim S. (2016). Designing Peptide Bunches on Nanocage for Bispecific or Superaffinity Targeting. Biomacromolecules.

[43] Georgiev I.S., Joyce M.G., Chen R.E., Leung K., McKee K., Druz A., Van Galen J.G., Kanekiyo M., Tsybovsky Y., Yang E.S. (2018). Two-Component Ferritin Nanoparticles for Multimerization of Diverse Trimeric Antigens. ACS Infect. Dis..

[44] Fan K., Jiang B., Guan Z., He J., Yang D., Xie N., Nie G., Xie C., Yan X. (2018). Fenobody: A Ferritin-Displayed Nanobody with High Apparent Affinity and Half-Life Extension. Anal. Chem..

[45] He D., Marles-Wright J. (2015). Ferritin family proteins and their use in bionanotechnology. New Biotechnol..

[46] Kanekiyo M., Wei C.-J., Yassine H.M., McTamney P.M., Boyington J.C., Whittle J.R.R., Rao S.S., Kong W.-P., Wang L., Nabel G.J. (2013). Self-assembling influenza nanoparticle vaccines elicit broadly neutralizing H1N1 antibodies. Nature.

[47] Qi M., Zhang X.-E., Sun X., Zhang X., Yao Y., Liu S., Chen Z., Li W., Zhang Z., Chen J. (2018). Intranasal Nanovaccine Confers Homo- and Hetero-Subtypic Influenza Protection. Small.

[48] Wei J., Li Z., Yang Y., Ma G., Su Z., Zhang S. (2020). An Apoferritin–Hemagglutinin Conjugate Vaccine with Encapsulated Nucleoprotein Antigen Peptide from Influenza Virus Confers Enhanced Cross Protection. Bioconjugate Chem..

[49] He L., De Val N., Morris C.D., Vora N., Thinnes T.C., Kong L., Azadnia P., Sok D., Zhou B., Burton D.R. (2016). Presenting native-like trimeric HIV-1 antigens with self-assembling nanoparticles. Nat. Commun..

[50] Saunders K.O., Wiehe K., Tian M., Acharya P., Bradley T., Alam S.M., Go E.P., Scearce R., Sutherland L., Henderson R. (2019). Targeted selection of HIV-specific antibody mutations by engineering B cell maturation. SScience.

[51] Wang W., Zhou X., Bian Y., Wang S., Chai Q., Guo Z., Wang Z., Zhu P., Peng H., Yan X. (2020). Dual-targeting nanoparticle vaccine elicits a therapeutic antibody response against chronic hepatitis B. Nat. Nanotechnol..

[52] Yan Y., Wang X., Lou P., Hu Z., Qu P., Li D., Li Q., Xu Y., Niu J., He Y. (2020). A nanoparticle-based hepatitis C virus vaccine with enhanced potency. J. Infect. Dis..

[53] Kanekiyo M., Bu W., Joyce M.G., Meng G., Whittle J.R., Baxa U., Yamamoto T., Narpala S., Todd J.-P., Rao S.S. (2015). Rational Design of an Epstein-Barr Virus Vaccine Targeting the Receptor-Binding Site. Cell.

[54] Zakeri B., Fierer J.O., Celik E., Chittock E.C., Schwarz-Linek U., Moy V.T., Howarth M. (2012). Peptide tag forming a rapid covalent bond to a protein, through engineering a bacterial adhesin. Proc. Natl. Acad. Sci. USA.

[55] Li Y., Leneghan D.B., Miura K., Nikolaeva D., Brian I.J., Dicks M.D.J., Fyfe A.J., Zakutansky S.E., De Cassan S., Long C.A. (2016). Enhancing immunogenicity and transmission-blocking activity of malaria vaccines by fusing Pfs25 to IMX313 multimerization technology. Sci. Rep..

[56] Jardine J., Julien J.-P., Menis S., Ota T., Kalyuzhniy O., McGuire A., Sok D., Huang P.-S., MacPherson S., Jones M. (2013). Rational HIV Immunogen Design to Target Specific Germline B Cell Receptors. Sci..

[57] Tokatlian T., Read B.J., Jones C.A., Kulp D.W., Menis S., Chang J.Y.H., Steichen J.M., Kumari S., Allen J.D., Dane E.L. (2018). Innate immune recognition of glycans targets HIV nanoparticle immunogens to germinal centers. Science.

[58] Allen M.D., Perham R.N. (1997). The catalytic domain of dihydrolipoyl acetyltransferase from the pyruvate dehydrogenase multienzyme complex of Bacillus stearothermophilus: Expression, purification and reversible denaturation. FEBS Lett..

[59] Domingo G.J., Orru’ S., Perham R.N. (2001). Multiple Display of Peptides and Proteins on a Macromolecular Scaffold Derived from a Multienzyme Complex. J. Mol. Biol..

[60] Caivano A., Doria-Rose N.A., Buelow B., Sartorius R., Trovato M., D’Apice L., Domingo G.J., Sutton W.F., Haigwood N.L., De Berardinis P. (2010). HIV-1 Gag p17 presented as virus-like particles on the E2 scaffold from Geobacillus stearothermophilus induces sustained humoral and cellular immune responses in the absence of IFNγ production by CD4+ T cells. Virolology.

[61] Neek M., Tucker J.A., Kim T.I., Molino N.M., Nelson E.L., Wang S.-W. (2018). Co-delivery of human cancer-testis antigens with adjuvant in protein nanoparticles induces higher cell-mediated immune responses. Biomaterials.

[62] Kedersha N.L., Rome L.H., Krotoski D.M., Domingo C., Bronner-Fraser M. (1986). Isolation and characterization of a novel ribonucleoprotein particle: Large structures contain a single species of small RNA. J. Cell Biol..

[63] Suprenant K.A. (2002). Vault Ribonucleoprotein Particles: Sarcophagi, Gondolas, or Safety Deposit Boxes?. Biochemistry.

[64] Kickhoefer V.A., Han M., Raval-Fernandes S., Poderycki M.J., Moniz R.J., Vaccari D., Silvestry M., Stewart P.L., Kelly K.A., Rome L.H. (2008). Targeting Vault Nanoparticles to Specific Cell Surface Receptors. ACS Nano.

[65] Han M., Kickhoefer V.A., Nemerow G.R., Rome L.H. (2011). Targeted Vault Nanoparticles Engineered with an Endosomolytic Peptide Deliver Biomolecules to the Cytoplasm. ACS Nano.

[66] Champion C.I., Kickhoefer V.A., Liu G., Moniz R.J., Freed A.S., Bergmann L.L., Vaccari D., Raval-Fernandes S., Chan A.M., Rome L.H. (2009). A Vault Nanoparticle Vaccine Induces Protective Mucosal Immunity. PLoS ONE.

[67] Kar U.K., Jiang J., Champion C.I., Salehi S., Srivastava M., Sharma S., Rabizadeh S., Niazi K., Kickhoefer V., Rome L.H. (2012). Vault Nanocapsules as Adjuvants Favor Cell-Mediated over Antibody-Mediated Immune Responses following Immunization of Mice. PLoS ONE.

[68] Jiang J., Liu G., Kickhoefer V.A., Rome L.H., Li L.-X., McSorley S.J., Kelly K.A. (2017). A Protective Vaccine against Chlamydia Genital Infection Using Vault Nanoparticles without an Added Adjuvant. Vaccines.

[69] Hsia Y., Bale J.B., Gonen S.G.D.S.T., Shi D., Sheffler W., Fong K.K., Nattermann Y.H.S.G.U., Xu C., Huang P.-S., Ravichandran R. (2016). Design of a hyperstable 60-subunit protein icosahedron. Nat. Cell Biol..

[70] Bruun T.U.J., Andersson A.-M.C., Draper S.J., Howarth M. (2018). Engineering a Rugged Nanoscaffold to Enhance Plug-and-Display Vaccination. ACS Nano.

[71] Bale J.B., Gonen S., Liu Y., Sheffler W., Ellis D., Thomas C., Cascio D., Yeates T.O., Gonen T., King N.P. (2016). Accurate design of megadalton-scale two-component icosahedral protein complexes. Science.

[72] Marcandalli J., Fiala B., Ols S., Perotti M., De Van Der Schueren W., Snijder J., Hodge E., Benhaim M., Ravichandran R., Carter L. (2019). Induction of Potent Neutralizing Antibody Responses by a Designed Protein Nanoparticle Vaccine for Respiratory Syncytial Virus. Cell.

[73] Brouwer P.J.M., Antanasijevic A., Berndsen Z., Yasmeen A., Fiala B., Bijl T.P.L., Bontjer I., Bale J.B., Sheffler W., Allen J.D. (2019). Enhancing and shaping the immunogenicity of native-like HIV-1 envelope trimers with a two-component protein nanoparticle. Nat. Commun..

[74] Rudra J.S., Sun T., Bird K.C., Daniels M.D., Gasiorowski J.Z., Chong A.S., Collier J.H. (2012). Modulating Adaptive Immune Responses to Peptide Self-Assemblies. ACS Nano.

[75] Zhang R., Smith J.D., Allen B.N., Kramer J.S., Schauflinger M., Ulery B.D. (2018). Peptide Amphiphile Micelle Vaccine Size and Charge Influence the Host Antibody Response. ACS Biomater. Sci. Eng..

[76] Rudra J.S., Mishra S., Chong A.S., Mitchell R.A., Nardin E.H., Nussenzweig V., Collier J.H. (2012). Self-assembled peptide nanofibers raising durable antibody responses against a malaria epitope. Biomaterials.

[77] Si Y., Wen Y., Kelly S.H., Chong A.S., Collier J.H. (2018). Intranasal delivery of adjuvant-free peptide nanofibers elicits resident CD8+ T cell responses. J. Control. Release.

[78] Gasiorowski J.Z., Collier J.H. (2011). Directed Intermixing in Multicomponent Self-Assembling Biomaterials. Biomacromolecules.

[79] Pompano R.R., Chen J., Verbus E.A., Han H., Fridman A., McNeely T., Collier J.H., Chong A.S. (2014). Titrating T-Cell Epitopes within Self-Assembled Vaccines Optimizes CD4+ Helper T Cell and Antibody Outputs. Adv. Heal. Mater..

[80] Mora-Solano C., Wen Y., Han H., Chen J., Chong A.S., Miller M.L., Pompano R.R., Collier J.H. (2017). Active immunotherapy for TNF-mediated inflammation using self-assembled peptide nanofibers. Biomaterials.

[81] Wen Y., Waltman A., Han H., Collier J.H. (2016). Switching the Immunogenicity of Peptide Assemblies Using Surface Properties. ACS Nano.

[82] Wu Y., Collier J.H. (2017). α-Helical coiled-coil peptide materials for biomedical applications. Wiley Interdiscip. Rev. Nanomed. Nanobiotechnology.

[83] Dobson C.M. (2003). Protein folding and misfolding. Nature.

[84] Wu Y., Norberg P.K., Reap E.A., Congdon K.L., Fries C.N., Kelly S.H., Sampson J.H., Conticello V.P., Collier J.H. (2017). A Supramolecular Vaccine Platform Based on α-Helical Peptide Nanofibers. ACS Biomater. Sci. Eng..

[85] Karch C.P., Li J., Kulangara C., Paulillo S.M., Raman S.K., Emadi S., Tan A., Helal Z.H., Fan Q., Khan M.I. (2017). Vaccination with self-adjuvanted protein nanoparticles provides protection against lethal influenza challenge. Nanomed. Nanotechnol. Biol. Med..

[86] Tao P., Zhu J., Mahalingam M., Batra H., Rao V.B. (2019). Bacteriophage T4 nanoparticles for vaccine delivery against infectious diseases. Adv. Drug Deliv. Rev..

[87] Leiman P.G., Kanamaru S., Mesyanzhinov V.V., Arisaka F., Rossmann M.G. (2003). Structure and morphogenesis of bacteriophage T4. Cell. Mol. Life Sci..

[88] Karam J.D., Karam J.D. (1994). Molecular Biology of Bacteriophage T4.

[89] Yang X., Lee J., Mahony E.M., Kwong P.D., Wyatt R., Sodroski J. (2002). Highly Stable Trimers Formed by Human Immunodeficiency Virus Type 1 Envelope Glycoproteins Fused with the Trimeric Motif of T4 Bacteriophage Fibritin. J. Virol..

[90] Du S.X., Idiart R.J., Mariano E.B., Chen H., Jiang P., Xu L., Ostrow K.M., Wrin T., Phung P., Binley J.M. (2009). Effect of trimerization motifs on quaternary structure, antigenicity, and immunogenicity of a noncleavable HIV-1 gp140 envelope glycoprotein. Virology.

[91] Ringe R.P., Yasmeen A., Ozorowski G., Go E.P., Pritchard L.K., Guttman M., Ketas T.A., Cottrell C.A., Wilson I.A., Sanders R.W. (2015). Influences on the Design and Purification of Soluble, Recombinant Native-Like HIV-1 Envelope Glycoprotein Trimers. J. Virol..

[92] Shrivastava T., Samal S., Tyagi A.K., Goswami S., Kumar N., Ozorowski G., Ward A.B., Chakrabarti B.K. (2018). Envelope proteins of two HIV-1 clades induced different epitope-specific antibody response. Vaccine.

[93] Li T., Zhang Z., Zhang Z., Qiao J., Rong R., Zhang Y., Yao Q., Li Z., Shen H., Huang F. (2019). Characterization of native-like HIV-1 gp140 glycoprotein expressed in insect cells. Vaccine.

[94] Krammer F., Margine I., Tan G.S., Pica N., Krause J.C., Palese P. (2012). A Carboxy-Terminal Trimerization Domain Stabilizes Conformational Epitopes on the Stalk Domain of Soluble Recombinant Hemagglutinin Substrates. PLoS ONE.

[95] Eggink D., Goff P.H., Palese P. (2013). Guiding the Immune Response against Influenza Virus Hemagglutinin toward the Conserved Stalk Domain by Hyperglycosylation of the Globular Head Domain. J. Virol..

[96] Lu Y., Welsh J.P., Swartz J.R. (2014). Production and stabilization of the trimeric influenza hemagglutinin stem domain for potentially broadly protective influenza vaccines. Proc. Natl. Acad. Sci. USA.

[97] Du L., Leung V.H.-C., Zhang X., Zhou J., Chen M., He W., Zhang H.-Y., Chan C.C.S., Poon V.K.-M., Zhao G. (2011). A Recombinant Vaccine of H5N1 HA1 Fused with Foldon and Human IgG Fc Induced Complete Cross-Clade Protection against Divergent H5N1 Viruses. PLoS ONE.

[98] Yu F., Li Y., Guo Y., Wang L., Yang J., Zhao G., Zhou Y., Du L., Jiang S. (2015). Intranasal vaccination of recombinant H5N1 HA1 proteins fused with foldon and Fc induces strong mucosal immune responses with neutralizing activity: Implication for developing novel mucosal influenza vaccines. Hum. Vaccines Immunother..

[99] McLellan J.S., Chen M., Joyce M.G., Sastry M., Stewart-Jones G.B.E., Yang Y., Zhang B., Chen L., Srivatsan S., Zheng A. (2013). Structure-Based Design of a Fusion Glycoprotein Vaccine for Respiratory Syncytial Virus. Sci..

[100] Stewart-Jones G.B.E., Thomas P.V., Chen M., Druz A., Joyce M.G., Kong W.-P., Sastry M., Soto C., Yang Y., Zhang B. (2015). A Cysteine Zipper Stabilizes a Pre-Fusion F Glycoprotein Vaccine for Respiratory Syncytial Virus. PLoS ONE.

[101] Tai W., Zhao G., Sun S., Guo Y., Wang Y., Tao X., Tseng C.-T.K., Li F., Jiang S., Lanying D. (2016). A recombinant receptor-binding domain of MERS-CoV in trimeric form protects human dipeptidyl peptidase 4 (hDPP4) transgenic mice from MERS-CoV infection. Virology.

[102] Kim E., Erdos G., Huang S., Kenniston T.W., Balmert S.C., Carey C.D., Raj V.S., Epperly M.W., Klimstra W.B., Haagmans B.L. (2020). Microneedle array delivered recombinant coronavirus vaccines: Immunogenicity and rapid translational development. EBioMedicine.

[103] Ren Z., Tian C., Zhu Q., Zhao M., Xin A., Nie W., Ling S., Zhu M., Wu J., Lan H. (2008). Orally delivered foot-and-mouth disease virus capsid protomer vaccine displayed on T4 bacteriophage surface: 100% protection from potency challenge in mice. Vaccine.

[104] Tao P., Mahalingam M., Zhu J., Moayeri M., Sha J., Lawrence W.S., Leppla S.H., Chopra A.K., Rao V.B. (2018). A Bacteriophage T4 Nanoparticle-Based Dual Vaccine against Anthrax and Plague. mBio.

[105] Tao Y., Strelkov S.V., Mesyanzhinov V.V., Rossmann M.G. (1997). Structure of bacteriophage T4 fibritin: A segmented coiled coil and the role of the C-terminal domain. Structure.

[106] Frank S., Kammerer R.A., Mechling D., Schulthess T., Landwehr R., Bann J., Guo Y., Lustig A., Bächinger H.P., Engel J. (2001). Stabilization of short collagen-like triple helices by protein engineering. J. Mol. Biol..

[107] Bhardwaj A., Walker-Kopp N., Wilkens S., Cingolani G. (2008). Foldon-guided self-assembly of ultra-stable protein fibers. Protein Sci..

[108] Yokoi N., Inaba H., Terauchi M., Stieg A.Z., Sanghamitra N.J., Koshiyama T., Yutani K., Kanamaru S., Arisaka F., Hikage T. (2010). Construction of Robust Bio-nanotubes using the Controlled Self-Assembly of Component Proteins of Bacteriophage T4. Small.

[109] Yang X., Farzan M., Wyatt R., Sodroski J. (2000). Characterization of Stable, Soluble Trimers Containing Complete Ectodomains of Human Immunodeficiency Virus Type 1 Envelope Glycoproteins. J. Virol..

[110] Fokine A., Chipman P.R., Leiman P.G., Mesyanzhinov V.V., Rao V.B., Rossmann M.G. (2004). Molecular architecture of the prolate head of bacteriophage T4. Proc. Natl. Acad. Sci. USA.

[111] Jiang J., Abu-Shilbayeh L., Rao V.B. (1997). Display of a PorA peptide from Neisseria meningitidis on the bacteriophage T4 capsid surface. Infect. Immun..

[112] Zhu J., Tao P., Mahalingam M., Sha J., Kilgore P., Chopra A.K., Rao V. (2019). A prokaryotic-eukaryotic hybrid viral vector for delivery of large cargos of genes and proteins into human cells. Sci. Adv..

